# Gag HIV‑1 Virus-like Particles and Extracellular Vesicles Functionalization with Spike Epitopes of SARS-CoV‑2 Using a Copper-Free Click Chemistry Approach

**DOI:** 10.1021/acs.bioconjchem.4c00559

**Published:** 2025-02-24

**Authors:** Marc García-Trujillo, Jesús Lavado-García, Arnau Boix-Besora, Francesc Gòdia, Laura Cervera

**Affiliations:** † Grup d’Enginyeria de Bioprocessos i Biocatàlisi Aplicada ENG4BIO, Escola d’Enginyeria, 16719 Universitat Autònoma de Barcelona, Campus de Bellaterra, Cerdanyola del Vallès, 08193 Barcelona, Spain; ‡ Novo Nordisk Foundation Center for Biosustainability, Technical University of Denmark, 2800 Kgs. Lyngby, Denmark; § 115336 Institut d’Investigació Biomèdica de Bellvitge—IDIBELL, L’Hospitalet de Llobregat, 08908 Barcelona, Spain

## Abstract

Enveloped nanoparticles such as extracellular vesicles (EVs) and virus-like particles (VLPs) have emerged as promising nanocarriers capable of transporting bioactive molecules for drug delivery and vaccination. Optimized functionalization methodologies are required to increase the functionalization levels of these nanoparticles, enhancing their performance. Here, a bioorthogonal copper-free strain-promoted azide–alkyne cycloaddition (SPAAC) reaction has been optimized to functionalize human immunodeficiency virus type 1 (HIV-1) Gag-based VLPs and EVs. The optimization process has been carried out through reaction kinetics and design of experiments (DoE) using Cy5 as a reporter molecule. The functionalization of both VLPs and EVs has been studied using super-resolution fluorescence microscopy (SRFM), revealing remarkable differences between Gag-VLPs and coproduced EVs. EVs produced by mock transfection and cell growth have been functionalized achieving a mean of 3618.63 ± 48.91 and 6498.75 ± 352.71 Cy5 molecules covalently linked per particle (Cy5_cov_/particle), respectively. Different nanoparticles have been functionalized with two linear B-cell epitopes from the Spike protein of SARS-CoV-2, S_315–338_ TSNFRVQPTESIVRFPNITNLCPF and S_648–663_ GCLIGAEHVNNSYECD, and analyzed by an immunoassay with sera from COVID-19 patients. The obtained results validate the selected B-cell epitopes and highlight the potential of the optimized functionalization approach for the development of nanoparticle-based vaccines.

## Graphical abstract

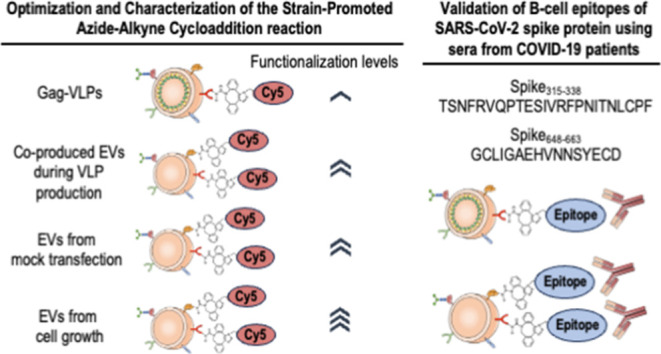

## 1 Introduction

The COVID-19 pandemic has evidenced the need for new platforms to rapidly generate multiple vaccine candidates, accelerating the initial stages of vaccine development as well as innovative methods for rapid vaccine manufacturing to cope with the potential emergence of new infectious disease outbreaks in the future. Extracellular vesicles (EVs), including exosomes and microvesicles, are cell-membrane-derived nanoparticles produced by cells containing proteins and nucleic acids such as mRNAs, miRNA, and DNA, among others.^1^ EVs allow cell-to-cell communication in a wide range of biological processes thanks to their capacity to selectively transport nucleic acids, proteins, and lipids.^1,2^ This characteristic has made them ideal nanoparticles to be used as nanocarriers, especially, in drug delivery^3,4^ and vaccination.^5,6^ Virus-like particles (VLPs) are nanoparticles that mimic the structural conformation of native viruses but lack viral genetic material, avoiding any possibility of reverse pathogenicity produced by infection or replication.^7^ VLPs of human immunodeficiency virus type 1 (HIV-1) are produced by the overexpression of Gag polyprotein, which can self-assemble in the inner leaflet of the cell membrane and form enveloped VLPs by means of a budding process. The plasmatic membrane embedding the Gag core of Gag-VLPs has been widely used to express different epitopes or viral proteins to target different diseases through coexpression.^8–13^ This functionalization approach based on gene transfection allows for the expression of only peptides or proteins on the plasmatic membrane that then are incorporated on the surface of VLPs and EVs during the budding process. Additionally, it is time-consuming and can present different challenges regarding transfection efficiency, recombinant protein expression, and protein incorporation into nascent nanoparticles, among others,^14^ causing that the whole process needs to be repeated and optimized for each peptide or protein.

Alternatively, the functionalization of EVs and VLPs can be achieved by chemical conjugation. This approach has been gaining interest recently thanks to the development of click chemistry. Click chemistry offers the advantage that it can be performed with EVs or VLPs already produced. This offers the possibility of having a VLP or EV stock fully purified and characterized, ready to be chemically modified to attach the desired epitopes, reducing the reaction time against novel outbreaks. Furthermore, the possibility of linking nonprotein molecules contributes to its versatility. A widely used click chemistry reaction is the copper-catalyzed alkyne–azide cycloaddition (CuAAC), but there is a harsh limitation with the use of copper, due to its toxicity.^15–19^ Azides and alkynes are nearly inert toward biological molecules^18,20–22^ and since they are not found in native biomolecules, this ensures specific functionalization.^21^ Strain-promoted alkyne–azide cycloaddition (SPAAC) arose from the need to eliminate the use of copper. Bertozzi and co-workers demonstrated that strained alkynes, such as cyclooctynes, activate the alkyne, avoiding the need of copper as a catalyst.^23^ Since SPAAC can be performed at physiological conditions,^24,25^ it has been widely used to functionalize exosomes and EVs, for many different purposes such as synergic cancer therapy,^26^ treatment of central nervous system injuries,^27^ drug delivery,^28–30^ antitumor vaccine,^31^ tissue engineering,^32^ and in vivo cells imaging.^33^ It has been also used with lentivirus,^34^ for enveloped virus labeling^35^ and exosome labeling,^36,37^ among other multiple applications. Nanoparticle functionalization with SPAAC can be performed basically through two main approaches, introducing azides or alkynes in biomolecules through metabolic incorporation of labeled metabolites^26,29,32,34,35,37–42^ or by direct chemical conjugation to the nanoparticles,^27,28,30,31,36^ allowing the orthogonal reaction with the partner.

Here, a SPAAC reaction is proposed to functionalize Gag-VLPs and the coproduced extracellular vesicles (EVs), as well as EVs alone. The functionalization pathway is a two-step reaction. In the first step, dibenzocyclooctyne-sulfo-*N*-hydroxysuccinimidyl ester (DBCO-sulfo-NHS) reacts with any primary amine present on the surface of VLPs or EVs. These primary amine are mainly lysines, one of the most abundant amino acid residues (6.3%).^43^ Lysines are usually solvent-exposed thanks to their hydrophilicity,^44^ making them an ideal target for high-density functionalization.^45^ In fact, lysines are one of the most common targeted amino acid residues,^46^ normally using NHS esters.^47^ This reaction forms an amide bond, allowing the DBCO to be exposed on the surface of the nanoparticles. In the second reaction step, azide-containing molecules react with the alkyne group of the DBCO to form a triazole linkage. Triazole linkages are highly water-soluble and have similar properties to amide bonds, but with different hydrolysis reactions, making them very stable in biological conditions.^48^ Additionally, its lack of flexibility hinders aggregation.^18^ In this work, the SPAAC reaction, previously reported for exosome functionalization,^28,31^ has been optimized to functionalize EVs achieving high epitope density to express epitopes of the SARS-CoV-2 Spike protein, and at the same time revealing valuable differences in membrane composition between Gag-VLPs and coproduced EVs.

## 2 Results

### 2.1 Functionalization of Virus-like Particles and Coproduced Extracellular Vesicles through a Strain-Promoted Azide–Alkyne Cycloaddition Reaction

The nanoparticle stock of VLPs and coproduced EVs was produced by transfecting a HEK293 cell culture with a plasmid encoding the Gag polyprotein of the HIV-1 virus fused in-frame with the enhanced green fluorescent protein (eGFP). In consequence, VLPs and EVs can be easily differentiated because VLPs contain Gag::eGFP monomers at the inner leaflet of the nanoparticle membrane, while the coproduced EVs lack Gag::eGFP monomers. At 72 h post-transfection, the supernatant of the transfected cell culture was harvested and ultracentrifugated using a 30% sucrose cushion to concentrate and purify VLPs and EVs from other proteins present in the supernatant, and at the same time enrich the percentage of VLPs with respect to total particles. The complete separation of VLPs from the coproduced EVs was not possible due to the similar size and physicochemical properties of these two types of nanoparticles. Despite this, the VLP + EV stock was composed by 71.52% of VLPs and 28.48% of EVs according to nanoparticle tracking analysis (NTA) (Figure S2A), which is a high ratio of VLP with respect to total particles considering reported data.^49^

As mentioned, the methodology used to functionalize VLPs and coproduced EVs is a bioorthogonal copper-free SPAAC reaction. By principle, the reaction pathway should be the same for VLPs and EVs, since both are embedded by the cellular lipidic membrane. During the first reaction step, DBCO-sulfo-NHS, that can link any primary amine-containing molecules present on the envelope of VLPs and EVs, is used as a bifunctional cross-linker. Then, during the second reaction step, azide-containing molecules can be covalently linked to the nanoparticles’ surface. After the second reaction step, there is an ultracentrifugation step to remove unincorporated reagents. Here, Cy5-azide is used as a reporter molecule to quantify nanoparticle functionalization, calculated as the average number of Cy5 molecules covalently linked per nanoparticle. The proof of concept for VLP and EV functionalization was carried out similarly to the approach described by Tian et al.^28^ However, the concentration of both reagents was balanced due to their equimolar ratio, as illustrated by the reaction scheme in Figure 1A. The overall binding achieved was 155.21 ± 18.35 Cy5 molecules per particle, with 76.7% of covalent attachment, which resulted in a mean of 116.53 ± 18.37 Cy5_cov_/particle (Cy5 molecules covalently linked per particle) (Figure 1B). It is possible that the proportion of Cy5 molecules not covalently bound could be attributed to electrostatic interactions, among other noncovalent interactions, or even binding resulting from steric hindrances, which may have hindered their removal during the ultracentrifugation step. To validate the covalent union, a Western blot was performed under denaturing conditions, demonstrating not only the covalent union of Cy5 molecules but also the promiscuity of the reaction that is able to link the molecule of interest to virtually any membrane protein or other amine-containing molecules (Figure 1C). This promiscuity is key to achieve high levels of epitope density. Different intensities of the bands observed in the Western blot also indicate that Cy5 is linked to a greater or lesser extent depending on the membrane protein, either because they contain a greater number of possible binding sites, have a higher concentration, or both. Furthermore, NTA and transmission electron microscopy (TEM) analysis showed that VLPs and coproduced EVs retained their typical morphology, suggesting that the functionalization process did not affect the integrity of the nanoparticles (Figures S2 and S3).

**Figure 1:**
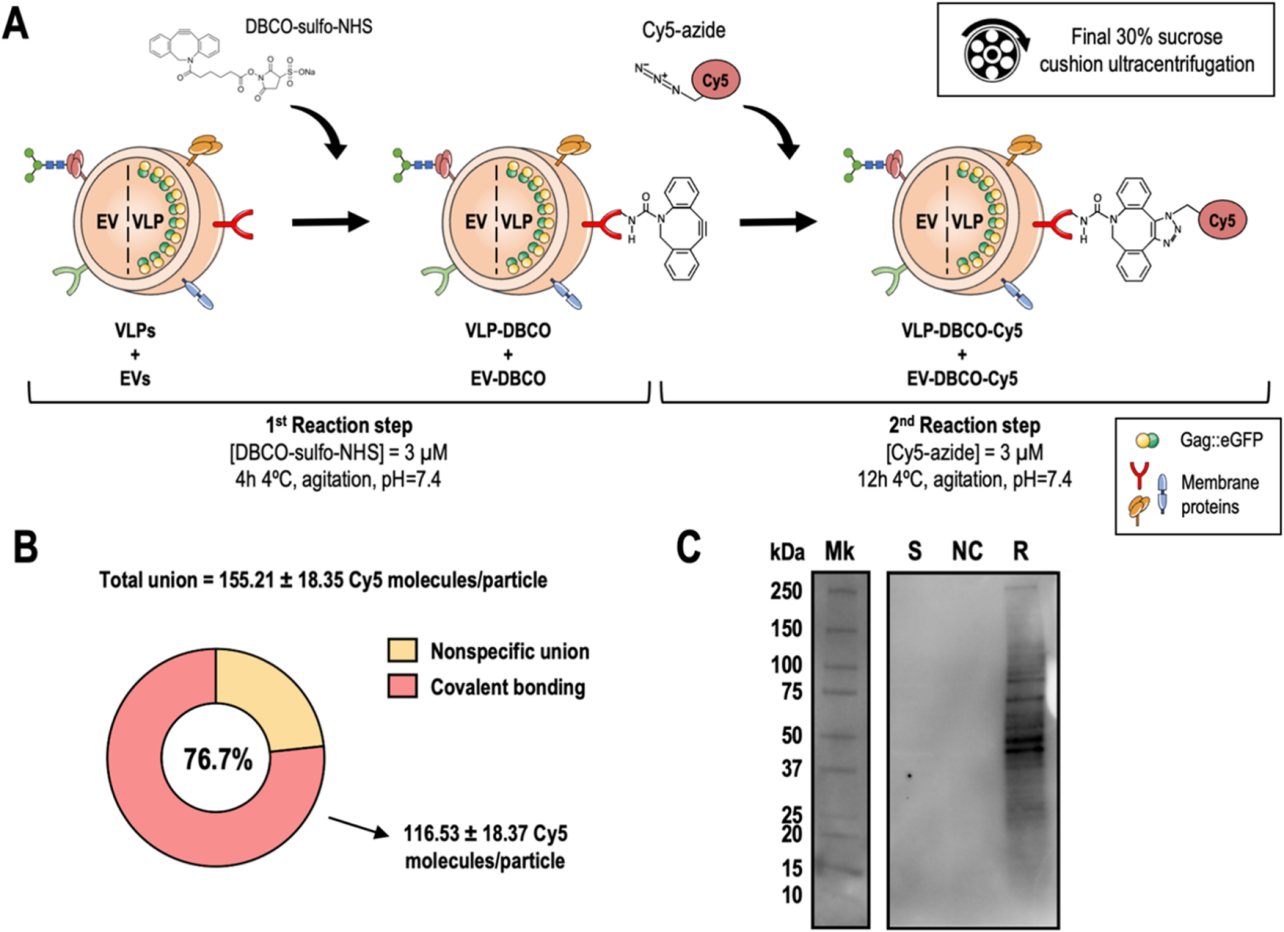
Proof of concept for functionalizing VLPs and coproduced EVs using a click chemistry approach. (A) Schematic representation of the proposed reaction to conjugate Cy5 fluorophore to the surface of VLPs and EVs by a two-step bioorthogonal copper-free strain-promoted azide–alkyne cycloaddition reaction. (B) Representation of the functionalization levels obtained. The values correspond to the mean ± standard deviation (SD); *n* = 3. (C) Western blot of the proof of concept for functionalizing VLPs and coproduced EVs. Mk: marker; S: VLP + EV stock; NC: negative control of the first reaction step corresponds to the reaction without the addition of DBCO-sulfo-NHS; R: reaction.

### 2.2 Optimization of SPAAC Reaction to Functionalize the VLP + EV Stock

In order to increase the functionalization levels obtained previously, an optimization process was performed. This was divided into two parts: first, determining the optimal reaction conditions (time and temperature) and, subsequently, optimizing the concentration ratios of nanoparticles and reagents. In Figure 2A, the kinetics of the first reaction step performed at two different temperatures (4 and 37 °C) is presented. There is an improvement only at 37 °C when the first reaction step is extended up to 20 or 24 h, reaching 274.72 Cy5 molecules covalently linked per nanoparticle at 24 h. Given the lack of significant differences between extending the first reaction step to 20 versus 24 h, a duration of 24 h was chosen to optimize and simplify the subsequent stages of the process. Additionally, the percentage of covalent union also increased up to 90.95%. It is also important to remark that neither the extension of the reaction time nor the temperature affected the nanoparticle concentration. As a result, the kinetic analysis for the second reaction step was conducted at 37 °C, preceded by a first reaction step performed for 24 h at 37 °C. The kinetics of the second reaction step (Figure 2B) shows no improvement in terms of functionalization levels when the reaction time is extended. Indeed, if the second reaction step is extended to 24 h, there is a decrease in the percentage of covalent bonding, indicating that extending the second reaction step promotes the nonspecific union of Cy5. Due to this, the second reaction step should be performed for 12 h at 37 °C.

**Figure 2:**
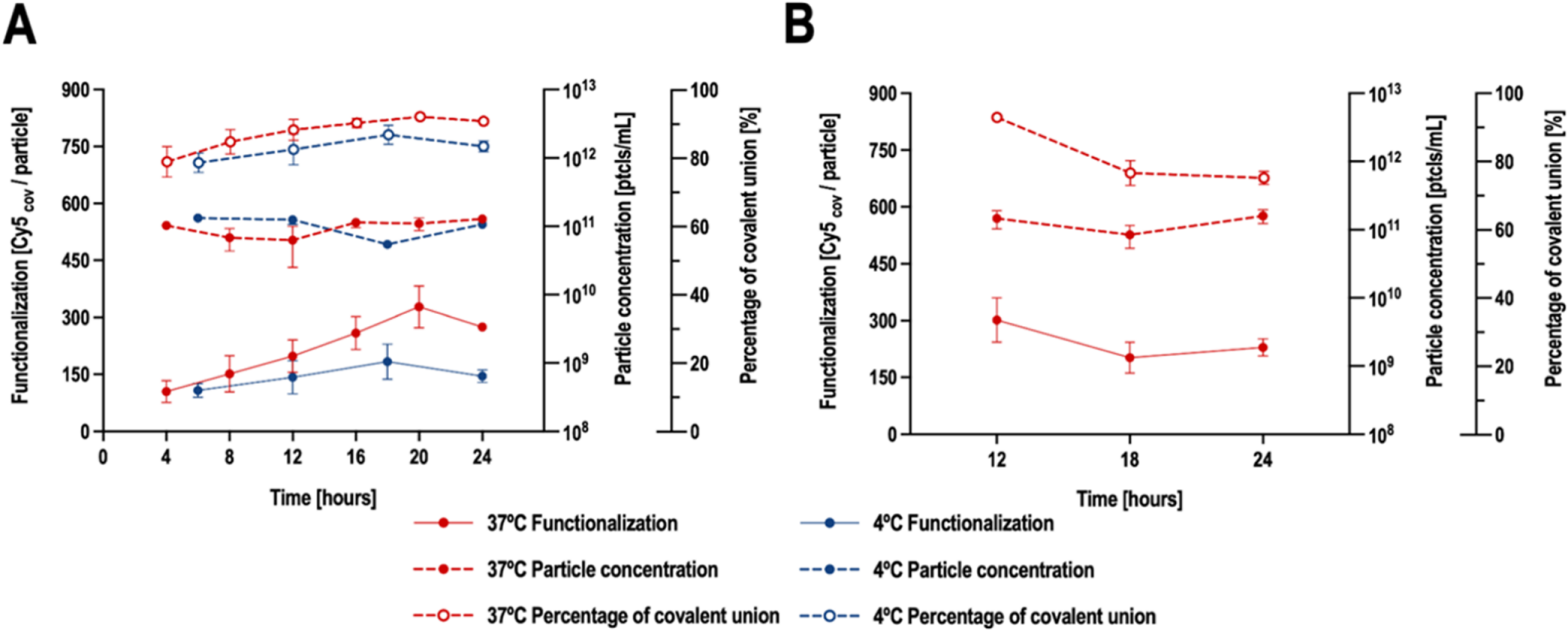
Kinetics of the two reaction steps of the strain-promoted azide–alkyne cycloaddition reaction. (A) Kinetics of the first reaction step performed at 4 and 37 °C. The reaction time of the second reaction step was maintained at 12 h at the corresponding temperature for all conditions. Error bars represent the standard deviation; *n* = 2. (B) Kinetics of the second reaction step performed at 37 °C. For all conditions, the first reaction step was carried out for 24 h at 37 °C. Error bars represent the standard deviation; *n* = 3.

Once the optimal reaction conditions were determined, a design of experiments (DoE) using a three-factor, three-level Box–Behnken design was conducted to optimize the nanoparticle concentration and the concentration of both reagents, DBCO-sulfo-NHS and Cy5-azide. Working ranges for each variable were selected according to preliminary experiments (data not shown). Nanoparticle concentration was limited between 3.5 × 10^11^ and 1.26 × 10^12^ ptcls/mL. DBCO-sulfo-NHS concentration was set up at a range of 10–100 μM. For Cy5-azide, the working range was set at 10–50 μM. Using these working ranges, a 15-experiment matrix was defined in which the central point was performed in triplicate. In addition, nine negative controls were performed, corresponding to all possible combinations of each nanoparticle concentration with each Cy5-azide concentration, in order to assess nonspecific Cy5 bonding. Experimental data were fitted to a second-order model nonlinear regression, using the covalent bonding of Cy5 per particle as response. The statistical significance of the model was confirmed by one-way analysis of variance (ANOVA), as shown in Table 1. The obtained results indicate the strong influence of nanoparticle and Cy5-azide concentration on nanoparticle functionalization. Nonetheless, since DBCO-sulfo-NHS is the reagent with less influence, it could be indicating that all primary amines present on the envelope of VLPs and EVs could be saturated by the union of DBCO-sulfo-NHS. Response surface graphs were constructed using the data obtained from the model (Figure 3). Since the model provided several similar optimal conditions, the one with the lowest Cy5-azide concentration was chosen to prevent nonspecific bonding and minimize the costs of the functionalization process, especially when applied to chemically synthesized peptides or other molecules. The optimal values with their corresponding coding levels for each variable are shown in Table 2. The optimal functionalization predicted value was 921.25 ± 79.21 Cy5 molecules covalently linked per particle. This optimal was experimentally validated with three independent replicates obtaining a functionalization value of 923.28 ± 59.26 Cy5 molecules covalently linked per particle with a 92.96% of covalent binding with respect to the total (Table 2). This means that considering all of the Cy5 present at the end of the reaction, only 7.04% of Cy5 was noncovalently bound and not fully removed. This percentage is consistent with the results from the kinetic study where a covalent binding efficiency of 90.95% was achieved, leaving 9.05% of noncovalently bound Cy5 that could not be entirely removed during ultracentrifugation. Thus, for Cy5, the increased concentration used does not appear to affect the efficiency of its removal. The DoE optimum supposes a 3.4-fold increase compared to the optimal value obtained after the kinetics studies, and a 7.9-fold increase compared to the initial proof of concept.

**Figure 3:**
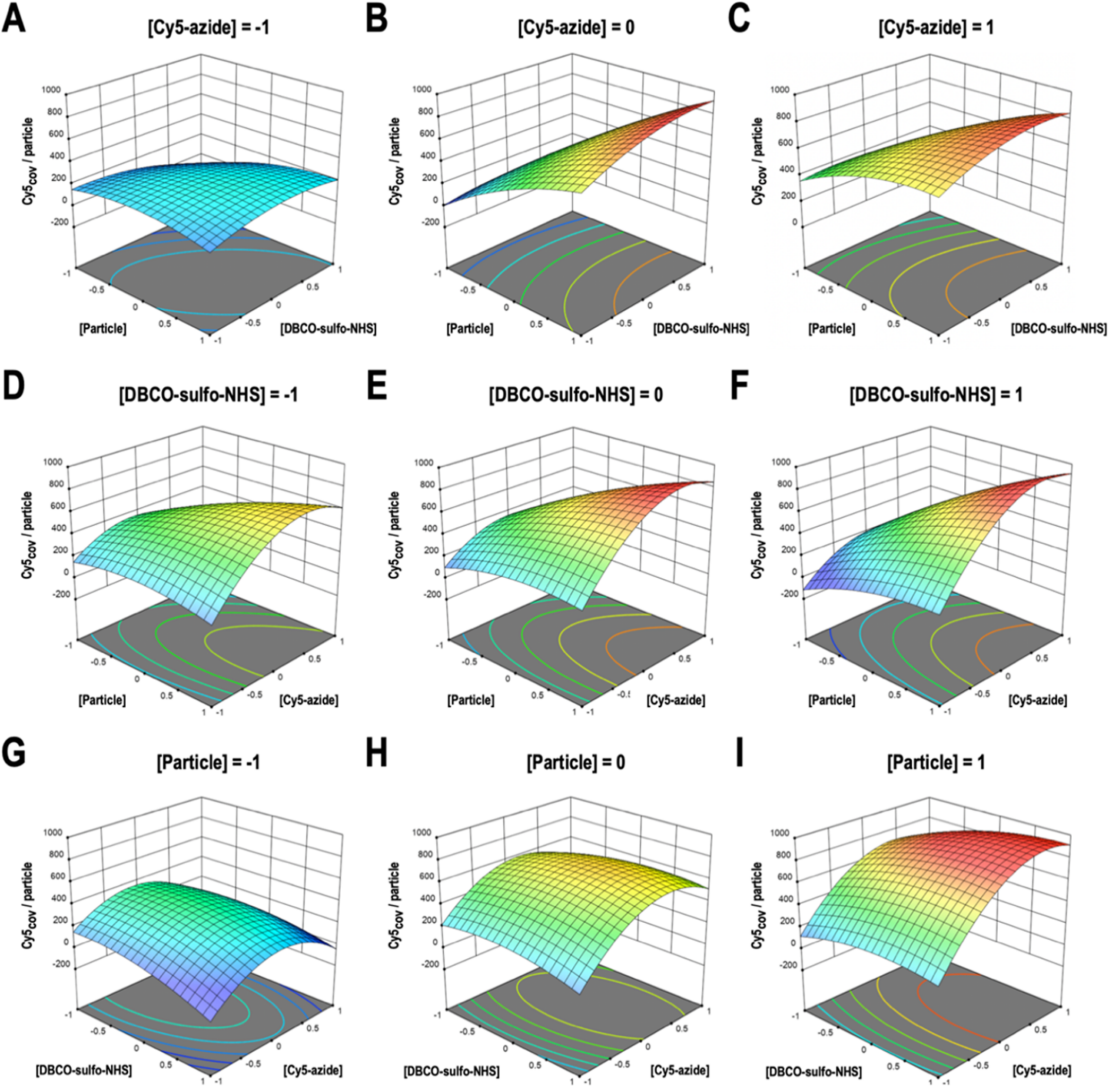
Response surface graphs from Box–Behnken experimental results. Functionalization levels measured as Cy5 molecules covalently linked per particle as a function of (A–C) particle concentration vs DBCO-sulfo-NHS concentration; (D–F) particle concentration vs Cy5-azide concentration; and (G–I) DBCO-sulfo-NHS concentration vs Cy5-azide concentration. The graphs were generated by representing two variables at a time maintaining the third variable at a constant level. −1, 0, and 1 coding levels correspond to 10, 30, and 50 μM for Cy5-azide concentration; 10, 55, and 100 μM for DBCO-sulfo-NHS concentration; and 3.50 × 10^11^, 8.04 × 10^11^, and 1.26 × 10^12^ ptcls/mL for particle concentration.

**Table 1:** Box–Behnken Design, Results, and ANOVA Analyses for Optimization of the Concentration of Total Particles, DBCO-sulfo-NHS, and Cy5-azide for Nanoparticle Functionalization

	coding levels		
parameters	–1	0	1
particle concentration [ptcls/mL]	3.50 × 10^11^	8.04 × 10^11^	1.26 × 10^12^
DBCO-sulfo-NHS concentration [μM]	10	55	100
Cy5-azide concentration [μM]	10	30	50

	coding levels			
experimental run	[particle]	[DBCO-sulfo-NHS]	[Cy5-azide]	Cy5_cov_/particle
1	0	0	0	560.42
2	1	0	1	849.28
3	0	0	0	691.80
4	–1	–1	0	342.88
5	0	–1	1	407.54
6	1	–1	0	627.65
7	–1	0	1	51.50
8	0	0	0	797.64
9	0	1	–1	103.53
10	–1	1	0	224.78
11	–1	0	–1	97.20
12	0	–1	–1	233.82
13	1	1	0	861.41
14	0	1	1	498.35
15	1	0	–1	248.48

ANOVA analyses				
*R* ^2^	adjusted *R* ^2^	predicted *R* ^2^	*p* value^a^	lack of fit^b^
0.9717	0.9207	0.8974	0.0023	0.9685

parameters	coefficient	*F*-value	*p* value
constant	683.29		
[particle]	233.81	69.70	0.0004
[DBCO-sulfo-NHS]	9.52	0.12	0.7477
[Cy5-azide]	140.46	25.25	0.0041
[particle]·[DBCO-sulfo-NHS]	87.97	4.93	0.0770
[particle]·[Cy5-azide]	161.63	16.65	0.0095
[DBCO-sulfo-NHS]·[Cy5-azide]	55.27	1.95	0.2216
[particle]^2^	–84.15	4.17	0.0967
[DBCO-sulfo-NHS]^2^	–84.96	4.25	0.0943
[Cy5-azide]^2^	–287.52	48.65	0.0009

a *p* values under 0.05 are considered statistically significant with 95% of confidence.

b A lack of fit above 0.05 indicates that the hypothesis arguing that the model is suitable cannot be rejected.

**Table 2:** Validation of the Predicted Optimal Conditions of the Box–Behnken Design

particle concentration [ptcls/mL]	DBCO-sulfo-NHS concentration [μM]	Cy5-azide concentration [μM]	predicted optimal value [Cy5_cov_/particle]	experimental optimal value [Cy5_cov_/particle]
1.22 × 10^12^ ptcls/mL	82.6 μM	35 μM	921.25 ± 79.21	923.28 ± 59.26
(0.967)^a^	(0.613)	(0.249)		92.96%^b^

a Values between brackets indicate the coding levels of each parameter.

b Percentage of covalent binding compared to the total.

### 2.3 Comparison of the Functionalizations of VLPs and EVs

The optimized reaction was characterized to validate nanoparticle functionalization and study the difference in functionalization between VLPs and EVs. To do so, as VLPs and EVs have a size that is around 100–200 nm (Figure S2), they were visualized using super-resolution fluorescence microscopy (SRFM). Some of the images obtained from SRFM are shown in Figure 4A–D. Here, the low presence of double-positive events can be observed, corresponding to either functionalized VLPs or instances where a VLP and a functionalized EV are in close proximity. As SRFM analysis allowed to determine the distance between the two peaks of intensity (Figure 4E,F), to remove false-positive events, all double-positive events with an intensity peak distance larger than 200 nm were considered as two different events, as a nonfunctionalized VLP and a functionalized EV, instead of being considered as a functionalized VLP. It is important to note that nonfunctionalized EVs could not be detected due to the lack of fluorescence. SRFM analysis revealed that only 4.45% of VLPs were Cy5-positive (Figure 4G). Moreover, 26.58% of the total observed events correspond to functionalized EVs. If these data are compared with the proportion of EVs relative to the total particles present in the sample, as determined by NTA (up to 26.43% as shown in Figure 4H), this indicates that nearly all EVs have been functionalized, a 99.4% if the obtained nanoparticle ratios are compared. According to these results, as functionalization levels were calculated as the average number of Cy5 molecules covalently linked per nanoparticle, these results evidence that EV present on the VLP + EV stock must have a more than 923.28 ± 59.26 Cy5_cov_/particle, and in the case of VLPs, these values must be lower. Despite the promiscuity of the proposed reaction, some differences in functionalization between both nanoparticles could be expected because it was already established that the membrane compositions of VLPs and EVs were already different, at least in terms of glycosylation, not only in the glycosylation pattern but also in the glycan density per particle.^50^ Despite this, the remarkable difference in functionalization between VLPs and coproduced EVs could be related not only to a higher concentration of membrane proteins in the envelope of EVs compared to VLPs but also to a different composition of membrane proteins for each coproduced nanoparticle. These valuable insights regarding the membrane composition between these two types of nanoparticles, which are similar in size and have very similar physicochemical properties, could be used in the future to develop new purification processes for the separation of VLPs from coproduced EVs, or improve the current purification protocols, to allow a better separation of both nanoparticles. In general, this characterization process points to EVs as the ideal nanoparticles to be functionalized through this click chemistry approach in comparison to VLPs.

**Figure 4:**
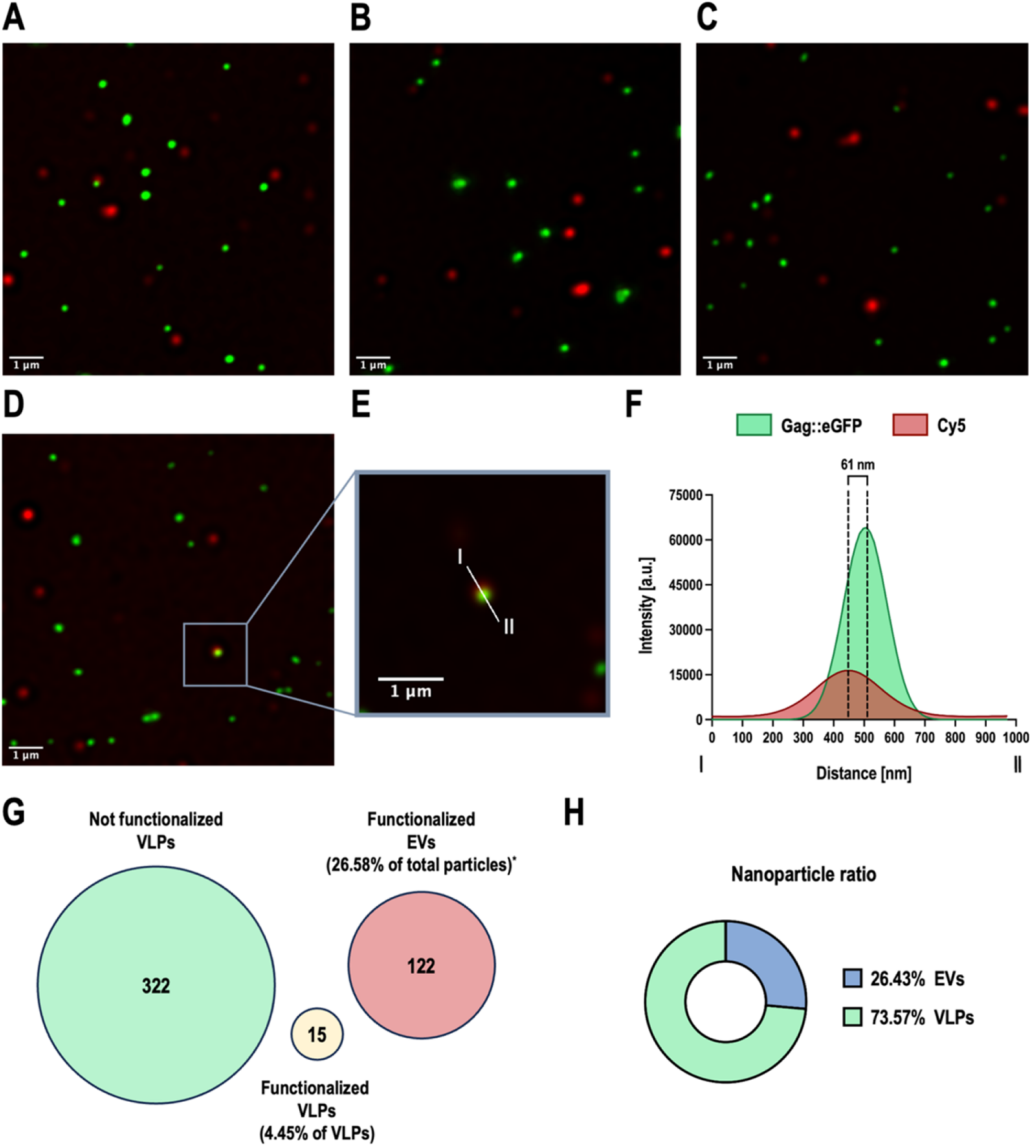
Functionalization analysis using super-resolution fluorescence microscopy. (A–D) Images of the DoE optimum obtained with SRFM. Gag::eGFP is colored green, while Cy5 is shown in red. (E) Enlargement of image (D), displaying the analyzed intensity profile. (F) Intensity profile of the line shown in image (E). The distance between the two intensities is shown. (G) Values obtained from the analysis of 16 different image fields from the DoE optimum. Each number represents the count of events detected in the analyzed images. The number in the green sphere represents GFP-positive events, corresponding to nonfunctionalized VLPs; the number in the red disk represents Cy5+ events, corresponding to functionalized EVs, and the number in the yellow disk represents GFP+ and Cy5+ events, corresponding to functionalized VLPs. To distinguish an aggregate from a VLP and a functionalized EV, all colocalizing green and red events with an intensity peak distance larger than 200 nm were considered two different events. *This percentage represents the functionalized EVs with respect to the total particles detected by SRFM. (H) Percentage of VLPs and EVs with respect to total particles according to NTA measurement of the DoE optimum sample that was used to perform the SRFM analysis.

Considering the high capability of extracellular vesicles to be functionalized through this click chemistry reaction, it was decided to generate two different stocks of EVs. The first one, produced by transient transfection utilizing a noncoding plasmid, to produce, as close as possible, similar EVs to those coproduced during VLP production (from now on called EV Mock stock). The other stock was produced solely through cell growth until the cell culture reached the plateau phase (called EV Growth stock). Once the EVs from each stock were produced and purified, they were functionalized by normalizing the nanoparticle concentration to the concentration of EVs present in the VLP + EV stock, equivalent to 3.5 × 10^11^ ptcls/mL. Both reagents, DBCO-sulfo-NHS and Cy5-azide, were used at the optimal concentrations obtained in the DoE. The functionalization levels as well as the percentage of covalent union obtained for each nanoparticle stock can be observed in Figure 5. Contrary to the VLP + EV stock, EV stocks showed some aggregation after the functionalization, as confirmed by NTA and TEM analysis (Figures S2 and S3). The extracellular vesicles from the Mock stock achieved a mean of 3618.63 ± 48.91 Cy5_cov_/particle. This result is in accordance with the SRFM analysis, which revealed that EVs were nearly all functionalized, but only 4.45% of VLPs were Cy5-positive. Indeed, if the result obtained in the DoE validation is divided only by the concentration of EVs instead of total particles, a mean of 3493.32 ± 224.25 Cy5_cov_/particle would be obtained, a value similar to what has been obtained with the EV Mock stock. This suggests that the EVs produced by mock transfection could have a similar membrane composition in terms of membrane proteins to those EVs that are coproduced during VLP production. In the case of the extracellular vesicles produced by cell growth, these are the highest functionalized nanoparticles achieving a mean of 6498.75 ± 352.71 Cy5_cov_/particle. This indicates that the EVs generated by cell growth are enriched in membrane proteins compared with the other nanoparticle stocks. Probably due to the lack of transfection and the extension of the production time, it is hypothesized that the cells have had more time to regenerate the membrane proteins present at the plasmatic membrane, and in consequence, the EVs generated are enriched in membrane proteins.

**Figure 5:**
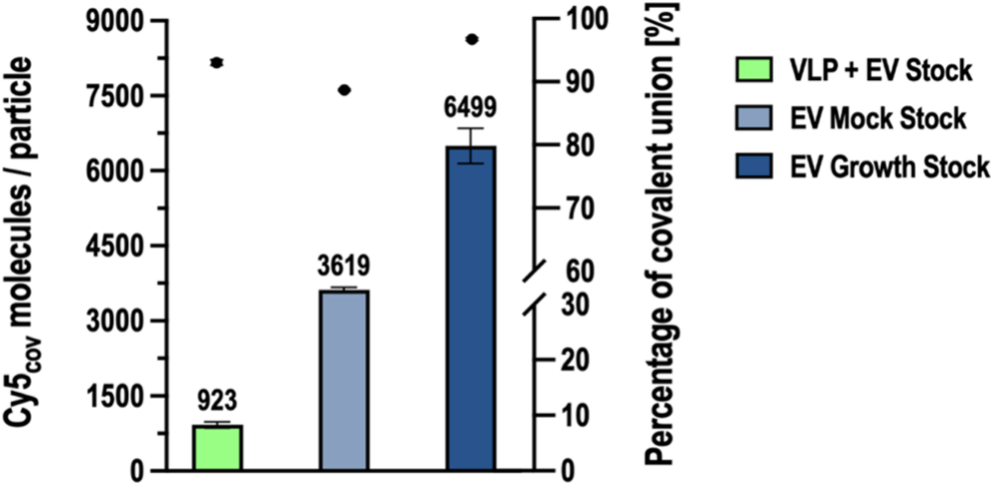
Functionalization of different nanoparticle stocks. The average of Cy5 molecules covalently linked per particle is represented in bars with the mean of the triplicate indicated at the top of the bar. The dots indicate the percentage of covalent union of each nanoparticle stock. Error bars represent the standard deviation; *n* = 3.

### 2.4 Immunoassay with Human Sera from COVID-19 Patients

Different nanoparticle stocks were functionalized with specific epitopes of SARS-CoV-2 to perform an immunoassay with sera from COVID-19 patients from Wuhan and Delta variants and assess the potential of utilizing SPAAC-based functionalization for the development of nanoparticle-based vaccines. The selected epitopes are linear epitopes from the Spike protein, predicted using Bepipred linear epitope prediction 2.0 method from the Immune Epitope Database and Analysis Resource using default parameters.^51^ This server reported a total of 34 possible B-cell epitopes from the Spike protein sequence, as described by Bhattacharya and co-workers.^52^ This list was reduced to 25 epitopes discarding sequences shorter than 8 amino acid residues in length (Table S1). After that, all epitopes were visualized using PyMOL molecular graphics systems (PDB: 6ZGE), and nonaccessible epitopes that were hidden inside the protein were discarded (Table S2). The resulting epitopes were compared with the literature, and finally two partially recognized epitopes were chosen. The selected predicted B-cell epitopes were S_315–338_ TSNFRVQPTESIVRFPNITNLCPF and S_648–663_ GCLIGAEHVNNSYECD, both peptide epitopes had been previously studied by Chen et al.^53^ Chen and co-workers determined these epitopes as good candidates for being B-cell epitopes, with high antigenicity scores, using a combination of the Bepipred linear epitope prediction 2.0 method and the ABC server.^53^ It is important to mention that the epitope S_315–338_ contains the beginning sequence of the receptor binding domain (RBD) of the Spike protein, from amino acids 331–338.^54^ When selecting B-cell epitopes, it is crucial to analyze the accessibility of the epitopes in relation to the glycan shield of the Spike protein because the high mobility of the glycan shield can potentially impede antibody recognition through steric hindrance.^55^ Both selected peptides have good accessibility considering the glycosylation of the Spike protein, even though the amino acid N_657_ is glycosylated, according to Grant et al.^56^ Different studies, which employed different bioinformatic tools along with the Bepipred linear epitope prediction 2.0 server either in combination with other servers or approaches, have identified and selected as potential B-cell epitopes, the peptides S_315–338_ ^55,57–61^ and S_648–663_ ^55,60,62–64^ either partially or entirely. Furthermore, the epitope S_648–663_ has been partially identified as a linear epitope of the Spike protein that exhibits higher recognition when tested against plasma from 12 convalescent COVID-19 patients.^63^ In addition, this epitope has been nearly completely recognized (S_649–663_) by IgG in an immunoassay employing sera from convalescent COVID-19 patients.^65^ For the epitope S_648–663_, the Omicron variant of SARS-CoV-2 contains a mutation compared to Delta and Wild-type variant of the virus at the amino acid 665, changing a histidine (H) for a tyrosine (Y).^66^ The amino acid H655 has been determined as an important amino acid for antibody recognition when it is replaced by an alanine,^63^ but it cannot be known whether the change to tyrosine also impedes recognition by an antibody or not. The impact of the H655Y mutation on immune system evasion could not be assessed as it is not possible to obtain sera solely from the Omicron variant. It cannot be discarded that COVID-19 patients have been previously exposed to an earlier variant of the SARS-CoV-2.

Different nanoparticle stocks were functionalized with either epitope S_315–338_ or S_648–663_, with an azide-containing lysine at the N-term of the epitope to make the SPAAC reaction possible. The reactions were performed with the previously optimized reaction parameters and reagent concentrations to perform the immunoassay with human sera from COVID-19 patients. The immunoassay was performed by incubating the sera with the following samples: nonfunctionalized nanoparticles, nanoparticles functionalized with the epitope S_315–338_ or S_648–663_, and negative controls from the first reaction step, where nanoparticles were treated without the addition of DBCO-sulfo-NHS to assess serum response to the nonspecifically bound epitopes. The antibody recognition from each serum for the various nanoparticle stocks is shown in Figure 6A for epitope S_315–338_ and in Figure 6B for epitope S_648–663_. As expected, negative sera did not recognize the epitopes attached in any nanoparticle stock. According to Figure 6A, all positive sera recognized the epitope attached to different nanoparticles, being the EV Mock stock and the VLP + EV stock, the stocks that exhibited a better antibody recognition (Figure 6C). In the case of S_648–663_, all positive sera recognized the epitope bound to the extracellular vesicles from EV Mock and EV Growth stock, with similar intensity, but not all sera recognized the epitope when it was presented by the VLP + EV stock. Considering that the immunoassay was performed using an equal concentration of nanoparticle from each stock, these results contrast with those obtained previously, where it was determined that nanoparticles from EV Growth stock exhibit a higher epitope density when functionalized with Cy5 (Figure 5). This could be explained by two hypotheses. It could be possible that functionalization levels could vary depending on the antigen to be linked and its physicochemical characteristics. This could be related to the difference observed in the percentage of covalent binding shown in Figure 6D. In the case of VLP + EV stock, the percentage of covalent binding decreases when the reaction is performed with the epitope S_648–663_, indicating also that the nonspecific union of the molecule of interest may vary also depending on its characteristics. On the other hand, it is also possible that depending on the antigen, there exists an optimal antigen density for recognition by antibodies. Exceeding this threshold may not enhance recognition, and an excessively high epitope density could potentially hinder proper recognition, leading to a counterproductive effect. Despite this, the fact that both epitopes were recognized by the majority of the sera with different nanoparticle stocks evidences that both epitopes are good B-cell epitope candidates for the potential development of a vaccine based on antigenic peptides presented by these nanoparticle stocks, especially with extracellular vesicles from EV Mock or EV Growth stock.

**Figure 6:**
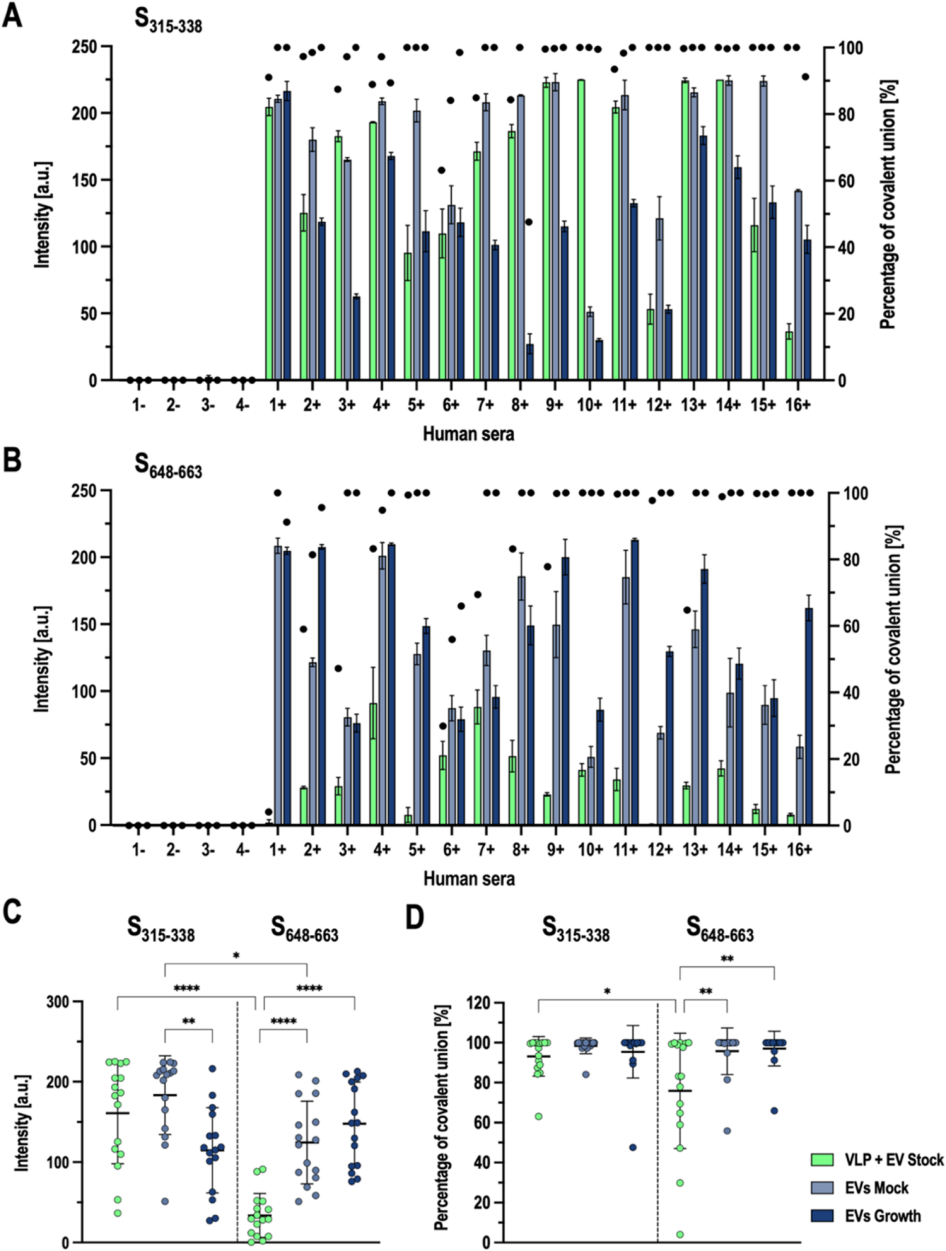
Immunoassay performed using human sera from COVID-19 patients. (A) Different bars represent the mean pixel intensity of each nanoparticle stock functionalized covalently with the epitope S_315–338_. Error bars indicate the standard deviation of the mean. Dots represent the percentage of covalent union obtained with each sera with different nanoparticle stocks. (B) Different bars represent the mean pixel intensity of each nanoparticle stock functionalized covalently with the epitope S_648–663_. Error bars indicate the standard deviation of the mean. Dots represent the percentage of covalent union obtained with each sera with different nanoparticle stocks. (C) Data points represent the mean intensity obtained for each individual sera with different nanoparticle stocks functionalized with each epitope. Bars represent the mean ± SD. (D) Data points represent the percentage of covalent union obtained for each individual sera with different nanoparticle stocks functionalized with each epitope. Bars represent the mean ± SD.

## 3 Conclusions

Conventionally, the functionalization of nanoparticles, such as exosomes or VLPs, was performed by using two main approaches: chemical conjugation or gene expression. In the gene expression method, viral proteins are typically expressed in cells, usually by transient transfection, so they are incorporated into vesicles or cotransfected with viral structural proteins, such as the HIV Gag polyprotein, to generate VLPs. In fact, the expression of epitopes or viral proteins to Gag-VLPs through gene transfection has been widely employed to target various diseases.^8–13^ However, this functionalization method often faces challenges related to the correct expression of recombinant proteins or the incorporation of these proteins into the EVs or VLPs, making it a time-consuming strategy.^14^ Given the transformative impact of click chemistry in recent decades and the need for rapid vaccine development methods, as highlighted by the COVID-19 pandemic, click chemistry functionalization has emerged as a promising alternative due to its speed and versatility. The optimized click chemistry methodology explored in this work enables easy and rapid functionalization of nanoparticles, achieving a high epitope density. One major time-saving factor is that different nanoparticle stocks, whether VLPs and coproduced EVs or just EVs produced through mock transfection or cell growth, can be already produced, purified, and stocked in advance to later link the antigenic epitopes of interest when required. Furthermore, the functionalization process can be performed in only 2 days, allowing a great reduction in time to be able to test different peptides or epitopes, since they can be easily changed. This is an advantage that can be used to accelerate the early stages of vaccine development. Moreover, another advantage of this approach is its versatility, since the nanoparticle can link basically any type of molecule as long as it contains an azide group, from a peptide to an antigenic epitope or another chemical molecule that could not be synthesized by a cell. The potential of this methodology could be used not only for vaccine development but also for drug delivery purposes, as it has been already demonstrated.^28–30^ An advantage of the optimized SPAAC method compared to other click chemistry approaches such as those based on CuAAC is that it does not require the use of copper, which has been reported as a toxic agent.^15–19^

One of the main drawbacks of this functionalization method is the need of an ultracentrifugation step to remove the unincorporated reagents, but for large productions or large-scale manufacturing the ultracentrifugation step could be substituted for an ultrafiltration or even a diafiltration, using tangential flow filtration (TFF), since these processes have been demonstrated to have no losses for VLPs and very small losses for EVs.^49^ In the case of diafiltration, this process can allow change of the reaction buffer to the final formulation buffer, prior to the use of the functionalized nanoparticles. In general, the characterization using SRFM has evidenced remarkable differences in membrane composition between VLPs and coproduced EVs, and has pointed EVs as the ideal nanoparticles to be functionalized through this click chemistry approach. This has been further evidenced with the high-density functionalization achieved with the EV Mock stock and EV Growth stock. When compared to similar works,^28,31^ this optimized SPAAC approach provides a notable advantage in achieving a high degree of functionalization. Additionally, the use of vesicles derived from cellular growth, which relies solely on cell growth without the need for DNA or transfection reagents, makes the process more economically viable, scalable, and straightforward. These advantages, combined with the positive results from the immunoassay, position these nanoparticles as excellent candidates for vaccine development via click chemistry. In summary, this work highlights the potential of the optimized SPAAC reaction for functionalizing EVs, paving the way for their use in vaccine development and drug delivery applications.

## 4 Materials and Methods

### 4.1 Cell Line and Culture Conditions

The cell line used is a serum-free suspension adapting the human embryonic kidney HEK293 cell line (HEK293 SF-3F6) from the National Research Council of Canada (NRC) (Montreal, Canada) kindly provided by Dr. Amine Kamen. Culture media was HyCell TransFx-H media from HyClone (GE Healthcare, Chicago, IL, USA) supplemented with 0.1% Pluronic F-68 Nonionic Surfactant (Gibco, Life Technologies, Thermo Fisher Scientific, Waltham, MA, USA) and 4 mM GlutaMAX (Gibco). Cell cultures were routinely maintained in an exponential growth phase with viabilities over 95% in 125 mL disposable polycarbonate shake flasks with a vent cap (Corning, New York, NY, USA) in an LT-X Kuhner shaker (LT-X Kuhner, Birsfelden, Switzerland) at 37 °C, 5% CO_2_, and 85% relative humidity at 130 rpm agitation. Cell density and viability were determined using a NucleoCounter NC-3000 automatic cell counter (Chemometec, Allerod, Denmark) according to manufacturer’s instructions.

### 4.2 Transient Transfection and Nanoparticle Stock Generation and Purification

Prior to transfection, cells were expanded in 1 L vented shake flasks (Corning) with a maximum cell culture volume of 200 mL per shake flask under previously described culture conditions. Transient transfections were performed at a cell density of 2 × 10^6^ viable cells/mL using PEIPro (PolySciences, Warrington, PA, USA) as a transfection reagent with a final DNA concentration of 1 μg/mL and a DNA to PEI mass ratio of 1:2. Plasmid DNA was diluted in fresh culture media (10% of culture volume to be transfected) and vortexed for 10 s. Then, PEI was added, and the mixture was vortexed three times for 3 s and incubated 15 min at room temperature (RT) to allow complex formation prior to its addition to the cell cultures.

VLPs and coproduced EVs (VLP + EV stock) were produced by transfecting 1.4 L of cell culture with a plasmid encoding a Rev-independent Gag polyprotein of the HIV-1 virus fused in-frame to the enhanced green fluorescent protein (eGFP). EVs produced by mock transfection (EV Mock stock) were produced by transfecting 0.9 L of cell culture with a mock plasmid that shares the same backbone as the plasmid coding for Gag::eGFP but has the CMV promoter changed by an SV40 terminator and lacks the ATG start codon. EVs secreted from cell growth (EV Growth stock) were produced from 0.6 L cell culture that was grown until reaching the plateau phase, achieving a cell density of 9 × 10^6^ viable cells/mL with a viability around 85%.

The supernatants of VLP + EV stock and EV Mock stock were harvested by culture centrifugation at 3000*g* for 10 min at 72 h post-transfection. Then, the supernatants were purified by 30% sucrose cushion ultracentrifugation at 164,000*g* for 2 h at 4 °C using a SW32-Ti rotor in an Optima L-100 XP ultracentrifuge (Beckman Coulter, Brea, CA, USA). Pellets were resuspended in prechilled phosphate-buffered saline (PBS) and stored at 4 °C overnight. Then, the resuspended pellets were homogenized, filtered using a 0.22 μm sterile filter to avoid future contamination, aliquoted, and stored at −80 °C until use.

The EV Growth stock was purified similar to a previously described protocol.^67^ Briefly, the supernatant of EV Growth stock was harvested by culture centrifugation at 3000*g* for 10 min. The supernatant was centrifuged at 10,000*g* for 10 min. Then, the obtained supernatant was centrifuged at 17,000*g* for 10 min. Finally, the resulting supernatant was ultracentrifuged as performed for the VLP + EV stock and EV Mock stock.

### 4.3 Nanoparticle Functionalization through Click Chemistry

Bioorthogonal strain-promoted azide–alkyne cycloaddition reactions were performed using a dibenzocyclooctyne-sulfo-*N*-hydroxysuccinimidyl ester (DBCO-sulfo-NHS) (Sigma, St. Louis, MO, USA) as a bifunctional cross-linker. During the first reaction step, DBCO-sulfo-NHS reacts with primary amine-containing molecules present on the surfaces of VLPs and EVs. Then, at the second reaction step, DBCO reacts with azide-containing molecules, forming a triazole bond that enables the molecule of interest to be covalently attached to VLP and EV surfaces. Reactions were performed in PBS at pH 7.4 without any catalyst, under agitation at 4 or 37 °C, as specified in each experiment. To remove unconjugated reagents, a 30% sucrose cushion ultracentrifugation at 164,000*g* for 2 h at 4 °C was performed using a SW32-Ti rotor in an Optima L-100 XP ultracentrifuge (Beckman Coulter). Ultracentrifugation supernatants were discarded, and functionalized nanoparticles were resuspended in prechilled PBS. Nanoparticle concentration and the concentration of each reagent are provided in the text for each particular condition. Cy5-azide (Merck, Darmstadt, Germany) was used as a reporter molecule to quantify nanoparticle functionalization, calculated as the average number of Cy5 molecules covalently linked per particle. Reaction kinetic studies were carried out by extending the time of the corresponding reaction step and maintaining the reaction time of the other reaction step at different temperatures with a DBCO-sulfo-NHS and Cy5-azide concentration of 3 μM. DoE experiments were performed at 37 °C for 24 h for the first reaction step and 12 h for the second reaction step. Nanoparticle functionalization with each epitope was performed according to the DoE optimal conditions.

### 4.4 Quantification of Cy5 by Fluorimetry

The intensity of Cy5 fluorescence in samples was measured by spectrofluorometry using a Cary Eclipse Fluorescence Spectrophotometer (Agilent Technologies, Santa Clara, CA, USA). The instrument parameters were set as follows to avoid scattering: λ_ex_ = 610 nm (slit 10 nm), λ_ex_ = 670 nm (slit 20 nm), and 0.1 s as average measurement time. Three technical replicates were measured per sample at room temperature. Relative fluorescence units (RFU) per nanoparticle values were calculated by subtracting fluorescent units (FU) per nanoparticle of negative control samples from that given by the sample. Correlation between fluorescence and Cy5 concentration was achieved by making a standard curve from a Cy5-azide sample of known concentration, obtaining a linear regression (Figure S1). Cy5 concentration was determined by eq 1, and the percentage of covalent bonding was calculated considering the amount of Cy5-azide associated with the negative control of the first reaction step

1 $$\mathrm{Cy}5‐\mathrm{azide concentration}\;\left[\mathrm{nM}\right]=\frac{\mathrm{RFUs}-3.8557}{12.7215}$$

### 4.5 HIV-1 Gag-VLPs Quantification through Flow Virometry

Quantification of Gag::eGFP VLPs by flow virometry was performed using a CytoFLEX LX (Beckman Coulter, Brea, CA, USA) with a violet side scatter (V-SSC) 405 nm filter configuration. The threshold of the area trigger signal fluorescein isothiocyanate (FITC) was set to 280. The laser gains were set as follows: 72 for FSC, 135 for SSC, 9 for V-SSC, and 500 for FITC. Samples were diluted in 0.22 μm filtered PBS in order to have between 500 and 5000 events/μL and an abort rate below 5%. A minimum of 20,000 VLP events per sample were recorded at a flow rate of 10 μL/min for further analysis using the CytExpert v.2.3 software (Beckman Coulter, Brea, CA, USA). V-SSC versus B525-FITC density plots were used to gate the VLP population from background noise. All measurements were normalized by an internal control to compare the results obtained from the nanoparticle tracking analysis.

### 4.6 HIV-1 Gag-VLPs and Total Nanoparticle Quantification by Nanoparticle Tracking Analysis (NTA)

Nanoparticle tracking analysis (NTA) was used to quantify the VLP and total nanoparticle concentration per reference. NTA measurements were carried out with a NanoSight NS300 (NanoSight Ltd., Amesbury, U.K.) equipped with a blue laser module (488 nm) to quantify Gag-VLPs and a neutral density filter to determine the total particle concentration by light scattering. Three technical replicate analyses were performed for each sample at room temperature. The obtained data were analyzed with NanoSight NTA 3.1 software.

### 4.7 Western Blot

Samples were loaded on sodium dodecyl sulfate-polyacrylamide gel electrophoresis (SDS-PAGE) and transferred onto a poly­(vinylidene difluoride) (PVDF) membrane using the Trans-Blot Turbo Transfer System (Bio-Rad, Hercules, CA, USA) following manufacturer’s instructions. To saturate nonused protein-binding sites, the membrane was blocked with 5% w/v nonfat dry milk PBS for 30 min at room temperature (RT) with gentle agitation and rinsed three times with 0.1% Tween-20 PBS. Then, the membrane was incubated overnight (O/N) at 4 °C under soft agitation with an anti-Cy5 mouse antibody (C1117, Merck) diluted 1:1000 in PBS. Then, three wash steps using 0.1% Tween-20 PBS were performed and the membrane was incubated 2 h at room temperature under agitation with an antimouse horseradish peroxidase (HRP) conjugated antibody diluted 1:5000 in PBS. Finally, the membrane was washed three times with 0.1% Tween-20 PBS, and revealed with enhanced chemiluminescence (ECL Clarity kit, Bio-Rad) using a Chemidoc MP (Bio-Rad).

### 4.8 Super-Resolution Fluorescence Microscopy (SRFM)

Nonfunctionalized VLPs and functionalized VLPs or EVs were observed with a LEICA TCS SP8 instrument (Leica Microsystems AG, Weztlar, Germany) equipped with a HyVolution module to enable super-resolution imaging. The excitation/emission parameters for GFP and Cy5 were set as follows: 488/510 nm, and 633/650–795 nm for GFP and Cy5, respectively. A 10 μL drop of sample was placed on a glass-mounted slide. The analysis was performed using Fiji ImageJ (SciJava, Open Source).^68^

### 4.9 Selection of Epitopes against SARS-CoV-2

Linear B-cell epitopes from the Spike protein (Uniprot ID: P0DTC2) of SARS-CoV-2 were identified using the Bepipred linear epitope prediction 2.0 method from the Immune Epitope Database and Analysis Resource. All linear epitopes shorter than 8 amino acid residues were automatically discarded. All epitopes were visualized using PyMOL molecular graphics systems (PDB: 6ZGE). Internal and nonaccessible epitopes were ruled out. The resulting epitopes were compared with those in the literature to ultimately select only two candidate epitopes for the immunoassay. The selected epitopes were chemically synthesized by the Institut de Química Avanzada de Catalunya (IQAC–CSIC, Barcelona, Spain), with a modified lysine containing an azide group added at the N-terminus of the peptide epitope. The N-terminus is acetylated and the C-terminus contains an amide group.

### 4.10 Immunoassay with Human Sera from COVID-19 Patients

The research procedure involving the use of human sera samples was approved by the Ethics Committee on Animal and Human Experimentation (CEEAH) (reference no. 5293) together with the Biosafety Committee (Reference No. HR-610-20) of Universitat Autnoma de Barcelona (UAB) meeting the ethical and legal requirements regarding research with human biological samples. Sera samples were provided by the Biobank of the Banc de Sang i Teixits (Barcelona, Spain). All samples were anonymized to remove any patient-specific information. A total of 20 sera samples were employed, with 4 negative and 16 positive. The four negative sera used as negative control belonged to noninfected and unvaccinated individuals. Positive sera were collected from COVID-19 patients infected with Wuhan or Delta variants (reverse transcription-quantitative polymerase chain reaction (RT-qPCR) confirmed). The procedure of this immunoassay is similar to that recently described.^8^ The immunoassay was performed by using a 96-well Bio-Dot Apparatus (Bio-Rad). Functionalized nanoparticles with different epitopes and controls were transferred into a nitrocellulose membrane by vacuum. The controls and samples used were as follows: nanoparticle stock (nonfunctionalized nanoparticles), nanoparticles functionalized with epitope S_315–338_, nanoparticles functionalized with epitope S_648–663_, and negative controls of the first reaction step for functionalized nanoparticles with each epitope. Specifically, these negative controls were nanoparticles that underwent the reaction without the addition of DBCO-sulfo-NHS, in order to assess the serum response to the nonspecifically bound epitopes. All samples were transferred at the same concentration, with the total amount of transferred nanoparticles being equal to 4.125 × 10^8^ particles per sample. The membrane was blocked with 5% w/v nonfat dry milk PBS for 1 h at room temperature with gentle agitation and rinsed three times with 0.1% Tween-20 PBS. Then, the membranes were incubated for 1 h at 4 °C under mild agitation with human serum diluted 1:500 in blocking buffer. Then, three washing steps with 0.1% Tween-20 PBS were performed and the membrane was incubated for 1 h at room temperature under agitation with an antihuman IgG (Fab specific)-HRP antibody produced in goat (#A0293, Sigma-Aldrich) diluted 1:10,000 in a blocking buffer. Finally, the membrane was washed three times with 0.1% Tween-20 PBS, and revealed with WesternSure PREMIUM Chemiluminescent Substrate (#926-95000, LI-COR Biosciences, Lincoln, NE, USA) in a Odyssey XF Imaging System (LI-COR Biosciences). Images were analyzed using Fiji ImageJ (SciJava, Open Source).^68^

### 4.11 Statistical Analysis

Statistical analysis was performed using GraphPad Prism v.9.5.0 (Graphpad Software, San Diego, CA, USA). Comparison between two groups was carried out by Student’s *t* test, and comparison between multiple groups was performed by one-way analysis of variance (ANOVA), considering α = 0.05. All experiments were conducted using three independent biological replicates, unless otherwise specified.

## Supplementary Material



## Notes

Standard curve of Cy5-azide; size distribution of different nanoparticle stocks measured by NTA; morphologic characterization by TEM; B-cell epitopes predicted using the BepiPred linear B-cell epitope prediction 2.0 method from IEDB; and predicted epitopes exposed on the surface of the Spike protein (PDF)

## Abbreviations

COVID-19: coronavirus disease
CuAAC: copper-catalyzed azide–alkyne cycloaddition
Cy5_cov_: Cy5 covalently linked
DBCO-sulfo-NHS: dibenzocyclooctyne-sulfo-NHS
DoE: design of experiments
eGFP: enhanced green fluorescent protein
EV: extracellular vesicle
Gag::eGFP: translational fusion of Gag and eGFP proteins
HEK293: human embryonic kidney 293 cells
HIV-1: human immunodeficiency virus type 1
Hpt: hours post-transfection
NTA: nanoparticle tracking analysis
O/N: overnight
PEI: polyethylenimine
Ptcls: particles
RFUs: relative fluorescent units
RT: room temperature
SARS-CoV-2: severe acute respiratory syndrome coronavirus 2
SPAAC: strain-promoted azide–alkyne cycloaddition
SRFM: super-resolution fluorescence microscopy
TEM: transmission electron microscopy
VLP: virus-like particle

## References

[1] Yáñez-Mó M., Siljander P. R. M., Andreu Z., Zavec A. B., Borràs F. E., Buzas E. I., Buzas K., Casal E., Cappello F., Carvalho J., Colás E., Cordeiro-Da Silva A., Fais S., Falcon-Perez J. M., Ghobrial I. M., Giebel B., Gimona M., Graner M., Gursel I., Gursel M., Heegaard N. H. H., Hendrix A., Kierulf P., Kokubun K., Kosanovic M., Kralj-Iglic V., Krämer-Albers E. M., Laitinen S., Lässer C., Lener T., Ligeti E., Line A., Lipps G., Llorente A., Lötvall J., Manček-Keber M., Marcilla A., Mittelbrunn M., Nazarenko I., Nolte-’t Hoen E. N. M., Nyman T. A., O’Driscoll L., Olivan M., Oliveira C., Pállinger É., Del Portillo H. A., Reventós J., Rigau M., Rohde E., Sammar M., Sánchez-Madrid F., Santarém N., Schallmoser K., Ostenfeld M. S., Stoorvogel W., Stukelj R., Van Der Grein S. G., Helena Vasconcelos M., Wauben M. H. M., De Wever O. (2015). Biological Properties
of Extracellular Vesicles and Their Physiological Functions. J. Extracell. Vesicles.

[2] Van
Niel G., D’Angelo G., Raposo G. (2018). Shedding Light on the
Cell Biology of Extracellular Vesicles. Nat.
Rev. Mol. Cell Biol..

[3] Herrmann I. K., Wood M. J. A., Fuhrmann G. (2021). Extracellular Vesicles as a Next-Generation
Drug Delivery Platform. Nat. Nanotechnol..

[4] Du S., Guan Y., Xie A., Yan Z., Gao S., Li W., Rao L., Chen X., Chen T. (2023). Extracellular Vesicles:
A Rising Star for Therapeutics and Drug Delivery. J. Nanobiotechnol..

[5] Sabanovic B., Piva F., Cecati M., Giulietti M. (2021). Promising
Extracellular Vesicle-Based Vaccines against Viruses, Including SARS-CoV-2. Biology.

[6] Santos P., Almeida F. (2021). Exosome-Based Vaccines: History, Current State, and
Clinical Trials. Front. Immunol..

[7] Donaldson B., Lateef Z., Walker G. F., Young S. L., Ward V. K. (2018). Virus-like
Particle Vaccines: Immunology and Formulation for Clinical Translation. Expert Rev. Vaccines.

[8] Boix-Besora A., Gòdia F., Cervera L. (2023). Gag Virus-like Particles Functionalized
with SARS-CoV-2 Variants: Generation, Characterization and Recognition
by COVID-19 Convalescent Patients’ Sera. Vaccines.

[9] Boix-Besora A., Lorenzo E., Lavado-García J., Gòdia F., Cervera L. (2022). Optimization, Production, Purification
and Characterization
of HIV-1 GAG-Based Virus-like Particles Functionalized with SARS-CoV-2. Vaccines.

[10] Venereo-Sanchez A., Simoneau M., Lanthier S., Chahal P., Bourget L., Ansorge S., Gilbert R., Henry O., Kamen A. (2017). Process Intensification
for High Yield Production of Influenza H1N1 Gag Virus-like Particles
Using an Inducible HEK-293 Stable Cell Line. Vaccine.

[11] Fontana D., Garay E., Cevera L., Kratje R., Prieto C., Gòdia F. (2021). Chimeric VLPs Based on HIV-1 Gag
and a Fusion Rabies
Glycoprotein Induce Specific Antibodies against Rabies and Foot-and-Mouth
Disease Virus. Vaccines.

[12] Chapman R., van Diepen M., Galant S., Kruse E., Margolin E., Ximba P., Hermanus T., Moore P., Douglass N., Williamson A. L., Rybicki E. (2020). Immunogenicity of HIV-1 Vaccines
Expressing Chimeric Envelope Glycoproteins on the Surface of Pr55
Gag Virus-Like Particles. Vaccines.

[13] Garay E., Fontana D., Villarraza J., Fuselli A., Gugliotta A., Antuña S., Tardivo B., Rodríguez M. C., Gastaldi V., Battagliotti J. M., Alvarez D., Castro E., Cassataro J., Ceaglio N., Prieto C. (2023). Design and Characterization
of Chimeric Rabies-SARS-CoV-2 Virus-like Particles for Vaccine Purposes. Appl. Microbiol. Biotechnol..

[14] Kim T. K., Eberwine J. H. (2010). Mammalian Cell Transfection:
The Present and the Future. Anal. Bioanal. Chem..

[15] Kennedy D. C., McKay C. S., Legault M. C. B., Danielson D. C., Blake J. A., Pegoraro A. F., Stolow A., Mester Z., Pezacki J. P. (2011). Cellular Consequences of Copper Complexes Used to Catalyze
Bioorthogonal Click Reactions. J. Am. Chem.
Soc..

[16] Mitra S., Keswani T., Dey M., Bhattacharya S., Sarkar S., Goswami S., Ghosh N., Dutta A., Bhattacharyya A. (2012). Copper-Induced Immunotoxicity Involves Cell Cycle Arrest
and Cell Death in the Spleen and Thymus. Toxicology.

[17] Meghani N. M., Amin H. H., Lee B. J. (2017). Mechanistic Applications
of Click
Chemistry for Pharmaceutical Drug Discovery and Drug Delivery. Drug Discovery Today.

[18] Hein C. D., Liu X. M., Wang D. (2008). Click Chemistry,
a Powerful Tool
for Pharmaceutical Sciences. Pharm. Res..

[19] Wang T., Guo Z. (2006). Copper in Medicine: Homeostasis, Chelation Therapy and Antitumor
Drug Design. Curr. Med. Chem..

[20] Wang Q., Chan T. R., Hilgraf R., Fokin V. V., Sharpless K. B., Finn M. G. (2003). Bioconjugation by
Copper­(I)-Catalyzed Azide-Alkyne
[3 + 2] Cycloaddition. J. Am. Chem. Soc..

[21] Ouyang T., Liu X., Ouyang H., Ren L. (2018). Recent Trends in Click Chemistry
as a Promising Technology for Virus-Related Research. Virus Res..

[22] Scinto S. L., Bilodeau D. A., Hincapie R., Lee W., Nguyen S. S., Xu M., am Ende C. W., Finn M. G., Lang K., Lin Q., Pezacki J. P., Prescher J. A., Robillard M. S., Fox J. M. (2021). Bioorthogonal Chemistry. Nat.
Rev. Methods Primers.

[23] Jewett J. C., Sletten E. M., Bertozzi C. R. (2010). Rapid Cu-Free
Click Chemistry with
Readily Synthesized Biarylazacyclooctynones. J. Am. Chem. Soc..

[24] Agard N. J., Prescher J. A., Bertozzi C. R. (2004). A Strain-Promoted
[3 + 2] Azide-Alkyne
Cycloaddition for Covalent Modification of Biomolecules in Living
Systems. J. Am. Chem. Soc..

[25] Agard N. J., Baskin J. M., Prescher J. A., Lo A., Bertozzi C. R. (2006). A Comparative
Study of Bioorthogonal Reactions with Azides. ACS Chem. Biol..

[26] Nie W., Wu G., Zhang J., Huang L., Ding J., Jiang A., Zhang Y., Liu Y., Li J., Pu K., Xie H. (2020). Responsive Exosome
Nano-bioconjugates for Synergistic Cancer Therapy. Angew. Chem..

[27] Ruan H., Li Y., Wang C., Jiang Y., Han Y., Li Y., Zheng D., Ye J., Chen G., Yang G. y., Deng L., Guo M., Zhang X., Tang Y., Cui W. (2023). Click Chemistry Extracellular
Vesicle/Peptide/Chemokine Nanocarriers
for Treating Central Nervous System Injuries. Acta Pharm. Sin. B.

[28] Tian T., Zhang H. X., He C. P., Fan S., Zhu Y. L., Qi C., Huang N. P., Xiao Z. D., Lu Z. H., Tannous B. A., Gao J. (2018). Surface Functionalized
Exosomes as Targeted Drug Delivery Vehicles
for Cerebral Ischemia Therapy. Biomaterials.

[29] Wang M., Altinoglu S., Takeda Y. S., Xu Q. (2015). Integrating Protein
Engineering and Bioorthogonal Click Conjugation for Extracellular
Vesicle Modulation and Intracellular Delivery. PLoS One.

[30] Haroon K., Zheng H., Wu S., Liu Z., Tang Y., Yang G. Y., Liu Y., Zhang Z. (2024). Engineered
Exosomes
Mediated Targeted Delivery of Neuroprotective Peptide NR2B9c for the
Treatment of Traumatic Brain Injury. Int. J.
Pharm..

[31] Zhu H., Wang K., Wang Z., Wang D., Yin X., Liu Y., Yu F., Zhao W. (2022). An Efficient and Safe MUC1-Dendritic
Cell-Derived Exosome Conjugate Vaccine Elicits Potent Cellular and
Humoral Immunity and Tumor Inhibition in Vivo. Acta Biomater..

[32] Lee C. S., Fan J., Hwang H. S., Kim S., Chen C., Kang M., Aghaloo T., James A. W., Lee M. (2023). Bone-Targeting Exosome
Mimetics Engineered by Bioorthogonal Surface Functionalization for
Bone Tissue Engineering. Nano Lett..

[33] Yoon H. I., Yhee J. Y., Na J. H., Lee S., Lee H., Kang S. W., Chang H., Ryu J. H., Lee S., Kwon I. C., Cho Y. W., Kim K. (2016). Bioorthogonal Copper
Free Click Chemistry for Labeling and Tracking of Chondrocytes In
Vivo. Bioconjugate Chem..

[34] Chu Y., Oum Y. H., Carrico I. S. (2016). Surface
Modification via Strain-Promoted
Click Reaction Facilitates Targeted Lentiviral Transduction. Virology.

[35] Huang L. L., Lu G. H., Hao J., Wang H., Yin D. L., Xie H. Y. (2013). Enveloped Virus Labeling via Both
Intrinsic Biosynthesis
and Metabolic Incorporation of Phospholipids in Host Cells. Anal. Chem..

[36] Xu L., Faruqu F. N., Liam-or R., Abu Abed O., Li D., Venner K., Errington R. J., Summers H., Wang J. T. W., Al-Jamal K. T. (2020). Design of Experiment
(DoE)-Driven in Vitro and in Vivo
Uptake Studies of Exosomes for Pancreatic Cancer Delivery Enabled
by Copper-Free Click Chemistry-Based Labelling. J. Extracell. Vesicles.

[37] Song S., Shim M. K., Lim S., Moon Y., Yang S., Kim J., Hong Y., Yoon H. Y., Kim I. S., Hwang K. Y., Kim K. (2020). In Situ One-Step
Fluorescence Labeling Strategy of Exosomes via Bioorthogonal
Click Chemistry for Real-Time Exosome Tracking in Vitro and in Vivo. Bioconjugate Chem..

[38] Lee T. S., Kim Y., Zhang W., Song I. H., Tung C. H. (2018). Facile Metabolic
Glycan Labeling Strategy for Exosome Tracking. Biochim. Biophys. Acta, Gen. Subj..

[39] Laomeephol C., Tawinwung S., Suppipat K., Arunmanee W., Wang Q., Amie Luckanagul J. (2024). Surface Functionalization
of Virus-like
Particles via Bioorthogonal Click Reactions for Enhanced Cell-Specific
Targeting. Int. J. Pharm..

[40] Zhao X., Shen Y., Adogla E. A., Viswanath A., Tan R., Benicewicz B. C., Greytak A. B., Lin Y., Wang Q. (2016). Surface Labeling of
Enveloped Virus with Polymeric Imidazole Ligand-Capped
Quantum Dots via the Metabolic Incorporation of Phospholipids into
Host Cells. J. Mater. Chem. B.

[41] Zhao X., Cai L., Adogla E. A., Guan H., Lin Y., Wang Q. (2015). Labeling of
Enveloped Virus via Metabolic Incorporation of Azido Sugars. Bioconjugate Chem..

[42] Oum Y. H., Desai T. M., Marin M., Melikyan G. B. (2016). Click Labeling of
Unnatural Sugars Metabolically Incorporated into Viral Envelope Glycoproteins
Enables Visualization of Single Particle Fusion. J. Virol. Methods.

[43] Gilis D., Massar S., Cerf N. J., Rooman M. (2001). Optimality of the Genetic
Code with Respect to Protein Stability and Amino-Acid Frequencies. Genome Biol..

[44] Schoonen L., Van Hest J. C. M. (2014). Functionalization
of Protein-Based Nanocages for Drug
Delivery Applications. Nanoscale.

[45] Smith M. T., Hawes A. K., Bundy B. C. (2013). Reengineering
Viruses and Virus-like
Particles through Chemical Functionalization Strategies. Curr. Opin. Biotechnol..

[46] Pokorski J. K., Steinmetz N. F. (2011). The Art
of Engineering Viral Nanoparticles. Mol. Pharmaceutics.

[47] Pickens C.
J., Johnson S. N., Pressnall M. M., Leon M. A., Berkland C. J. (2018). Practical
Considerations, Challenges, and Limitations of Bioconjugation via
Azide-Alkyne Cycloaddition. Bioconjugate Chem..

[48] Kolb H. C., Sharpless K. B. (2003). The Growing Impact of Click Chemistry on Drug Discovery. Drug Discovery Today.

[49] Lorenzo E., Miranda L., Gòdia F., Cervera L. (2023). Downstream Process
Design for Gag HIV-1 Based Virus-like Particles. Biotechnol. Bioeng..

[50] Lavado-García J., Zhang T., Cervera L., Gòdia F., Wuhrer M. (2022). Differential N- and
O-Glycosylation Signatures of HIV-1
Gag Virus-like Particles and Coproduced Extracellular Vesicles. Biotechnol. Bioeng..

[51] Jespersen M. C., Peters B., Nielsen M., Marcatili P. (2017). BepiPred-2.0:
Improving Sequence-Based B-Cell Epitope Prediction Using Conformational
Epitopes. Nucleic Acids Res..

[52] Bhattacharya M., Sharma A. R., Patra P., Ghosh P., Sharma G., Patra B. C., Lee S. S., Chakraborty C. (2020). Development
of Epitope-Based Peptide Vaccine against Novel Coronavirus 2019 (SARS-COV-2):
Immunoinformatics Approach. J. Med. Virol..

[53] Chen H. Z., Tang L. L., Yu X. L., Zhou J., Chang Y. F., Wu X. (2020). Bioinformatics Analysis
of Epitope-Based Vaccine Design against the
Novel SARS-CoV-2. Infect. Dis. Poverty.

[54] Jackson C. B., Farzan M., Chen B., Choe H. (2022). Mechanisms of SARS-CoV-2
Entry into Cells. Nat. Rev. Mol. Cell Biol..

[55] Sikora M., von Bülow S., Blanc F. E. C., Gecht M., Covino R., Hummer G. (2021). Computational Epitope Map of SARS-CoV-2 Spike Protein. PLoS Comput. Biol..

[56] Grant O. C., Montgomery D., Ito K., Woods R. J. (2020). Analysis
of the
SARS-CoV-2 Spike Protein Glycan Shield Reveals Implications for Immune
Recognition. Sci. Rep.

[57] Lin L., Ting S., Yufei H., Wendong L., Yubo F., Jing Z. (2020). Epitope-Based Peptide
Vaccines Predicted against Novel Coronavirus
Disease Caused by SARS-CoV-2. Virus Res..

[58] Polyiam K., Phoolcharoen W., Butkhot N., Srisaowakarn C., Thitithanyanont A., Auewarakul P., Hoonsuwan T., Ruengjitchatchawalya M., Mekvichitsaeng P., Roshorm Y. M. (2021). Immunodominant Linear
B Cell Epitopes in the Spike and Membrane Proteins of SARS-CoV-2 Identified
by Immunoinformatics Prediction and Immunoassay. Sci. Rep..

[59] Can H., Köseoğlu A. E., Erkunt Alak S., Güvendi M., Döşkaya M., Karakavuk M., Gürüz A.
Y., Ün C. (2020). In Silico
Discovery of Antigenic Proteins and Epitopes of SARS-CoV-2 for the
Development of a Vaccine or a Diagnostic Approach for COVID-19. Sci. Rep..

[60] Vashi Y., Jagrit V., Kumar S. (2020). Understanding the B and T Cell Epitopes
of Spike Protein of Severe Acute Respiratory Syndrome Coronavirus-2:
A Computational Way to Predict the Immunogens. Infect., Genet. Evol..

[61] He J., Huang F., Zhang J., Chen Q., Zheng Z., Zhou Q., Chen D., Li J., Chen J. (2021). Vaccine Design
Based on 16 Epitopes of SARS-CoV-2 Spike Protein. J. Med. Virol..

[62] Pourseif M. M., Parvizpour S., Jafari B., Dehghani J., Naghili B., Omidi Y. (2021). A Domain-Based
Vaccine Construct against SARS-CoV-2, the Causative
Agent of COVID-19 Pandemic: Development of Self-Amplifying MRNA and
Peptide Vaccines. BioImpacts.

[63] Farrera-Soler L., Daguer J. P., Barluenga S., Vadas O., Cohen P., Pagano S., Yerly S., Kaiser L., Vuilleumier N., Winssinger N. (2020). Identification
of Immunodominant Linear Epitopes from
SARS-CoV-2 Patient Plasma. PLoS One.

[64] Wang D., Mai J., Zhou W., Yu W., Zhan Y., Wang N., Epstein N. D., Yang Y. (2020). Immunoinformatic
Analysis of T-and
B-Cell Epitopes for SARS-CoV-2 Vaccine Design. Vaccines.

[65] Schwarz T., Heiss K., Mahendran Y., Casilag F., Kurth F., Sander L. E., Wendtner C. M., Hoechstetter M. A., Müller M. A., Sekul R., Drosten C., Stadler V., Corman V. M. (2021). SARS-CoV-2 Proteome-Wide Analysis
Revealed Significant
Epitope Signatures in COVID-19 Patients. Front.
Immunol..

[66] Kumar S., Thambiraja T. S., Karuppanan K., Subramaniam G. (2022). Omicron and
Delta Variant of SARS-CoV-2: A Comparative Computational Study of
Spike Protein. J. Med. Virol..

[67] Pérez-Rubio P., Lavado-García J., Bosch-Molist L., Romero E. L., Cervera L., Gòdia F. (2024). Extracellular Vesicle Depletion and UGCG Overexpression
Mitigate the Cell Density Effect in HEK293 Cell Culture Transfection. Mol. Ther.—Methods Clin. Dev..

[68] Schneider C. A., Rasband W. S., Eliceiri K. W. (2012). NIH Image
to ImageJ: 25 Years of
Image Analysis. Nat. Methods.

