# Supplementary material for: Gag HIV‑1 Virus-like Particles and Extracellular Vesicles Functionalization with Spike Epitopes of SARS-CoV‑2 Using a Copper-Free Click Chemistry Approach
Source: Bioconjug Chem. 2025 Feb 24;36(3):486–99. doi: 10.1021/acs.bioconjchem.4c00559 (PMC12123617; doi:10.1021/acs.bioconjchem.4c00559)
Supplement: Supplementary file 1 [file bc4c00559_si_001.pdf]

Supporting Information

Gag HIV-1 Virus-Like Particles and Extracellular Vesicles functionalization with Spike epitopes of SARS-CoV-2 using a copper-free click chemistry approach

Marc García-Trujillo[†]*, Jesús Lavado-García[†‡], Arnau Boix-Besora[†§], Francesc Gòdia[†], Laura Cervera[†]*

[†]Grup d'Enginyeria de Bioprocessos i Biocatàlisi Aplicada ENG4BIO, Escola d'Enginyeria, Universitat Autònoma de Barcelona, Campus de Bellaterra, Cerdanyola del Vallès, 08193 Barcelona, Spain

[‡]Novo Nordisk Foundation Center for Biosustainability, Technical University of Denmark, 2800 Kgs. Lyngby, Denmark

[§]Institut d'Investigació Biomèdica de Bellvitge - IDIBELL, L'Hospitalet de Llobregat, 08908 Barcelona, Spain

Corresponding authors. E-mail adresses: marc.garciat@uab.cat (M. García-Trujillo), laura.cervera@uab.cat (L. Cervera)

Table of content

Figure S1. Standard curve of Cy5-azide……………………………………………………………………………3

Figure S2. Size distribution of the different nanoparticle stocks measured by NTA……….…………………4

Figure S3. Morphologic characterization by TEM…………………………………..………………..……5

Table S1. B-cell epitopes predicted using the Bepipred linear B cell epitope prediction 2.0 method from IEDB..…. ……………..……………..…………….……………………..………….…..……….……6

Table S2. Predicted epitopes exposed on the surface of the Spike protein. ……….……………….…….7

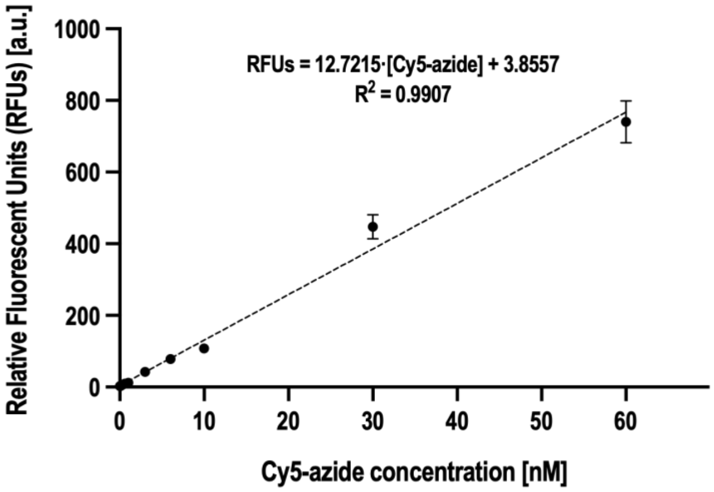

Figure S1. Standard curve of Cy5-azide. Correlation between relative fluorescent units (RFUs) and Cy5-azide concentration. Error bars represent the standard error of the mean calculated as $\varepsilon = SD/\sqrt{n}$, being $n = 3$. The curve was obtained by serial dilutions in PBS of a Cy5-azide sample of known concentration. The RFUs of each point were pared with their corresponding concentration, resulting in the linear regression shown.

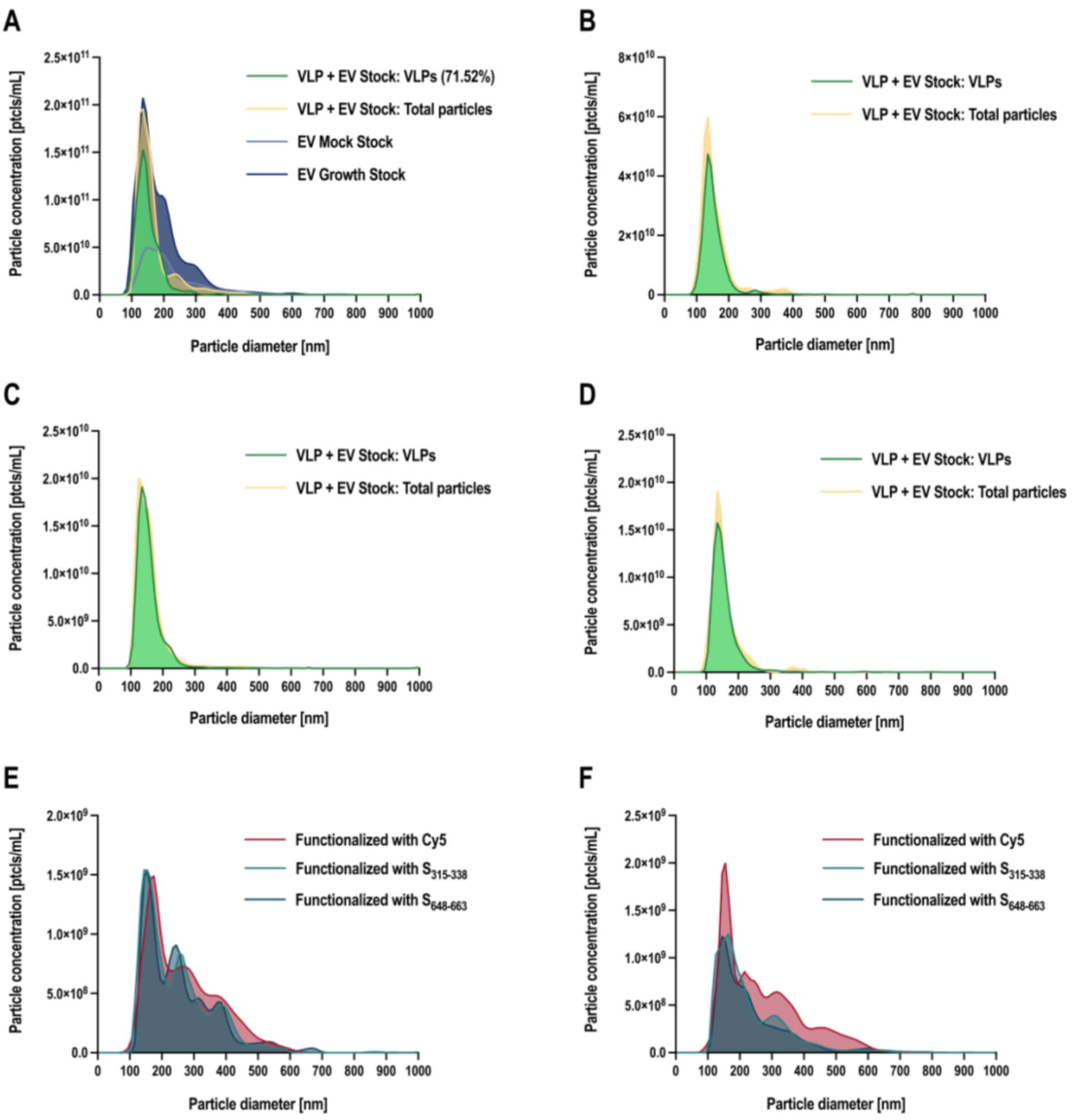

Figure S2. Size distribution of the different nanoparticle stocks measured by NTA. A) NTA measurement of the different nanoparticle stocks prior its functionalization. The value in brackets indicates the percentage of VLPs respect total particles of the VLP + EV stock. **B)** NTA measurement of the VLP + EV stock functionalized with Cy5. **C)** NTA measurement of the VLP + EV stock functionalized with epitope $S_{315-338}$. **D)** NTA measurement of the VLP + EV stock functionalized with epitope $S_{648-663}$. **E)** NTA measurements of the EV Mock stock functionalized with Cy5, epitope $S_{315-338}$ and epitope $S_{648-663}$. **F)** NTA measurements of the EV Growth stock functionalized with Cy5, epitope $S_{315-338}$ and epitope $S_{648-663}$.

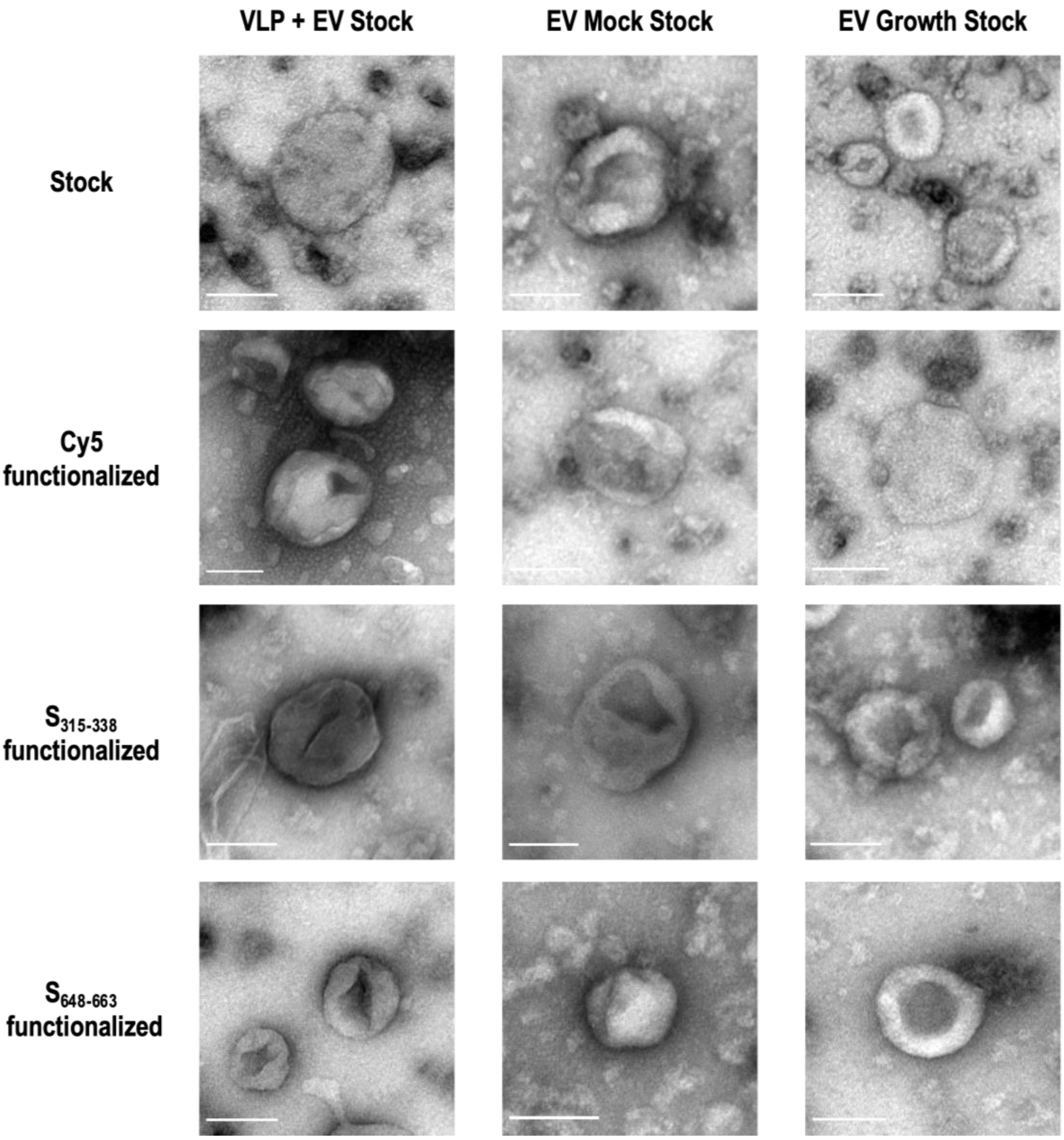

Figure S3. Morphologic characterization by TEM. It is shown the different nanoparticles unfunctionalized, and functionalized with Cy5, epitope $S_{315\text{-}338}$ and epitope $S_{648\text{-}663}$. Scale bar represents 100 nm.

Table S1. B-cell epitopes predicted using the Bepipred linear B cell epitope prediction 2.0 method from IEDB.

Start	End	Epitope sequence	Length*	Mean Score
13	37	SQCVNLTTRTQLPPAYTNSFTRGVY	25	586.60
59	81	FSNVTWFHAIHVSGTNGTKRFDN	23	552.87
138	154	DPFLGVYYHKNNKSWME	17	567.71
177	189	MDLEGKQGNFKNL	13	543.69
206	221	KHTPINLVRDLPQGFS	16	583.25
250	260	TPGDSSSGWTA	11	589.36
304	322	KSFTVEKGIYQTSNFRVQP	19	541.74
329	363	FPNITNLCPFGEVFNATRFASVYAWNRKRISNCVA	35	565.17
369	393	YNSASFSTFKCYGVSPTKLNDLCFT	25	543.52
404	426	GDEVRQIAPGQTGKIADYNYKLP	23	568.30
440	501	NLDSKVGGNYNYLYRLFRKSNLKPFERDISTEIYQAGSTPCNGVE GFNCYFPLQSYGFQPTN	62	551.16
516	536	ELLHAPATVCGPKKSTNLVKN	21	557.10
555	562	SNKKFLPF	8	552.63
616	632	NCTEVPVAIHADQLTPT	17	540.00
634	644	RVYSTGSNVFQ	11	524.18
656	666	VNNSYECDIPI	11	564.55
672	690	ASYQTQTNSPRRARSVASQ	19	625.42
695	710	YTMSLGAENSVAYSNN	16	613.19
786	800	KQIYKTPPIKDFGGF	15	561.60
807	814	PDPSKPSK	8	567.63
828	842	LADAGFIKQYGDCLG	15	528.47
1035	1043	GQSKRVDFC	9	527.33
1107	1118	RNFYEPQIITTD	12	537.33
1133	1172	VNNTVYDPLQPELDSFKEELDKYFKNHTSPDVDLGDISGI	40	559.80
1252	1267	SCCKFDEDDSEPVLKG	16	544.31

*All predicted epitopes shorter than 8 aminoacidic residues have been discarded as possible B-cell epitope candidates.

Table S2. Predicted epitopes exposed on the surface of the Spike protein.

Start	End	Sequence	Length	Mean Score*
13	37	SQCVNLTTRTQLPPAYTNSFTRGVY	25	586.60
138	154	DPFLGVYYHKNNKSWME	17	567.71
177	189	MDLEGKQGNFKNL	13	543.69
206	221	KHTPINLVRDLPQGFS	16	583.25
250	260	TPGDSSSGWTA	11	589.36
315	322	TSNFRVQP[a]	8	553.38
329	363	FPNITNLCPFGEVFNATRFASVYAWNRKRISNCVA	35	565.17
440	451	NLDSKVGGNYNY[b]	12	567.17
471	501	IYQAGSTPCNGVEGFNCYFPLQSYGFQPTN[b]	31	549.61
526	536	GPKKSTNLVKN	11	564.09
555	562	SNKKFLPF	8	552.63
634	644	RVYSTGSNVFQ	11	524.18
656	666	VNNSYECDIPI	11	564.55
695	710	YTMSLGAENSVAYSNN	16	613.19
807	814	PDPSKPSK	8	567.63
828	842	LADAGFIKQYGDCLG	15	528.47

*The mean score of the modified epitopes has been recalculated according to their new sequences.

[a]Amino acids 304-314 (KSFTVEKGIYQ) have been removed as they were not exposed on the surface of the Spike protein.

[b]Amino acids 452-470 (LYRLFRKSNLKPFERDISTE) have been removed as they were not exposed on the surface of the Spike protein. Consequently, the initial predicted epitope has been divided into two different epitopes.
